# Climate change impact uncertainty assessment and adaptations for sustainable maize production using multi-crop and climate models

**DOI:** 10.1007/s11356-021-17050-z

**Published:** 2021-10-27

**Authors:** Mubashra Yasin, Ashfaq Ahmad, Tasneem Khaliq, Muhammad Habib-ur-Rahman, Salma Niaz, Thomas Gaiser, Iqra Ghafoor, Hafiz Suboor ul Hassan, Muhammad Qasim, Gerrit Hoogenboom

**Affiliations:** 1 Sugarcane Research Institute, Ayub Agricultural Research Institute, Faisalabad, Pakistan; 2 Asian Disaster Preparedness Centre (ADPC), Islamabad, Pakistan; 3 Agro-Climatology Lab, Department of Agronomy, University of Agriculture, Faisalabad, Pakistan; 4 Institute of Crop Science and Resource Conservation (INRES), University Bonn, 53115 Bonn, Germany; 5 Department of Agronomy, MNS-University of Agriculture Multan, Multan, 60650 Pakistan; 6 Sera Processing Lab, National Institute of Health, Islamabad, Pakistan; 7 Department of Economics, Finance and Statistics, Jönköping University, Jönköping, Sweden; 8 Institute for Sustainable Food Systems, University of Florida, 184 Rogers Hall, Gainesville, FL 32611 USA

**Keywords:** Sowing time, Maize hybrids, CERES-Maize, CSM-IXIM, APSIM-Maize, Phenology, LAI, TDM, Yield, Climate variability, Adaptation, Sustainable maize production

## Abstract

Future climate scenarios are predicting considerable threats to sustainable maize production in arid and semi-arid regions. These adverse impacts can be minimized by adopting modern agricultural tools to assess and develop successful adaptation practices. A multi-model approach (climate and crop) was used to assess the impacts and uncertainties of climate change on maize crop. An extensive field study was conducted to explore the temporal thermal variations on maize hybrids grown at farmer’s fields for ten sowing dates during two consecutive growing years. Data about phenology, morphology, biomass development, and yield were recorded by adopting standard procedures and protocols. The CSM-CERES, APSIM, and CSM-IXIM-Maize models were calibrated and evaluated. Five GCMs among 29 were selected based on classification into different groups and uncertainty to predict climatic changes in the future. The results predicted that there would be a rise in temperature (1.57–3.29 °C) during the maize growing season in five General Circulation Models (GCMs) by using RCP 8.5 scenarios for the mid-century (2040–2069) as compared with the baseline (1980–2015). The CERES-Maize and APSIM-Maize model showed lower root mean square error values (2.78 and 5.41), higher *d*-index (0.85 and 0.87) along reliable *R*^2^ (0.89 and 0.89), respectively for days to anthesis and maturity, while the CSM-IXIM-Maize model performed well for growth parameters (leaf area index, total dry matter) and yield with reasonably good statistical indices. The CSM-IXIM-Maize model performed well for all hybrids during both years whereas climate models, NorESM1-M and IPSL-CM5A-MR, showed less uncertain results for climate change impacts. Maize models along GCMs predicted a reduction in yield (8–55%) than baseline. Maize crop may face a high yield decline that could be overcome by modifying the sowing dates and fertilizer (fertigation) and heat and drought-tolerant hybrids.

## Introduction

Current production systems in the world are most vulnerable to climate change (IPCC 2014; Rosenzweig et al. 2014; Rahman et al. 2018; Wiebe et al. 2019; Shafqat et al, 2019). Changing climate is expected to decrease crop production and ultimately constitutes a threat to food security especially in arid to semi-arid climatic regions (Zhang et al. 2007; Ahmad et al. 2015; Ahmed et al. 2018a). Temperature increase and uncertain rainfall patterns have adverse impacts on crops’ developmental phases, growth, and yield, and these impacts are already more pronounced in arid regions (Abbas et al. 2017; Ahmad et al. 2019; Ullah et al. 2019; Chattha et al. 2021). Variation in the climatic projections is most important in climate change studies for adoptions in crop production systems. Climatic changes include long-term variations in temperature, fluctuations in rainfall distributions, rising levels of CO_2_ and other atmospheric gases, and a rise in the existence of acute weather events (IPCC 2014, 2021; Porter 2005; Shafqat et al. 2021).

Maize is an important crop for food security, especially in developing countries. Demand is increased due to its numerous usages of domestic, commercial, and industrial as bio-fuel (Khaliq et al. 2008; Rosegrant et al. 2012; Asseng et al. 2014). However, maize production is adversely susceptible to extreme weather events due to climatic variability (Ahmed et al. 2018b). Changes in temperature especially elevated conditions have the potential to offset the optimum growth and development and shorten the growing seasons and ultimately reduced the yield (Yasin et al. 2019). Physiological and metabolic processes occurring in maize require optimum climatic conditions for proper growth and development (Wahid et al. 2007; Hatfield and Prueger 2015). Variation in optimum temperature ranges leads to a reduction in maize production; especially, these hazardous effects speed up under high day and night temperatures (Soler et al. 2007; Taylor et al. 2012; Yasin et al. 2019). The majority of the maize genotypes grown in these regions are extremely prone to raise temperature and drought stress. Crop genotypes with the ability to use water more efficiently can be used to cope with the water shortage conditions (Mubeen et al. 2020). As reproductive phases (flowering and grain-filling) are more vulnerable to high temperature, determination of optimum sowing time is essential for intensification and diversification in the current cropping system for sustainability (Mubeen et al. 2016; Ahmed et al. 2018a, b). Future projections show that there might be a rise of temperature up to 2.8 °C by 2069 in Pakistan (Ahmad et al. 2015). Among cereals crops getting successful maize production is under threat due to heat stress and less availability of other crucial resources like water and nutrients due to rising temperature (Lobell et al. 2011; Babel et al. 2019). Elevated CO_2_ has some positive effects on growth and yield, but maize being a C4 crop might have less advantage in photosynthetic accumulation for final biomass production (Ghannoum et al. 2000; Mina et al. 2019). Furthermore, it has also been reported in studies regarding future climate scenarios that interactive effects of CO_2_, projected temperature rise, and variability in rainfall could potentially reduce the positive effect of increasing CO_2_ concentration (Lobell et al. 2011; Hatfield and Prueger 2015). The predictions of unexpected periodic spells of heat stress are projected to occur more frequently in the region. These variations in climatic conditions are potentially altering the phases of maize phenology, growth, development, and yield and are a serious threat to sustainable maize production in the region and ultimately a threat to food security.

Crop growth models are innovative tools to determine the impacts of crop management practices like sowing dates, and plant genetics, and even interaction of these with the environment (Jones et al. 2003; Saddique et al. 2020a, b, c). Previously, these tools were used to assess climate change effects on crop production like effects of high temperature, elevated carbon dioxide, and uneven rainfall patterns (Lobell et al. 2011; Asseng et al. 2014). Various modeling techniques help determine the information about short-term and long-term management practices and production technologies for better crop production (Aurbacher et al. 2013). The Decision Support System for Agro-technology Transfer (DSSAT) is a complete package of system analysis approach having evaluation techniques of crop management including planting dates, irrigation, nutrients, and many others (Jones et al. 2003; Hoogenboom 2000; Hoogenboom et al. 2019). It has the potential to simulate the impacts and interactions of soil, plant genetics, and atmospheric variables on crop development, growth, and yield in various regions (Jones et al. 2003; Soler et al. 2007; Rahman et al. 2019; Saddique et al. 2019). The CSM-CERES-Maize under DSSAT model has been evaluated for maize crop management like irrigation, water, and nitrogen (Mubeen et al. 2016; Ahmed et al. 2018a, b; Ahmad et al. 2019). Furthermore, these are also being used to assess the impact of climate change on maize production under different climate change scenarios (Lobell et al. 2011; Mangani et al. 2019). Further, structural development under CERES-Maize was made related to leaf area, grain number, cob growth, integration and partitioning, and yield for better simulation and ultimately resulted in CSM-IXIM-Maize model under the DSSAT (Lizaso et al. 2011). Better predictions regarding crop phenology are of prime importance to evaluate the changing climate effects on crop yield, as uncertainties in the simulations of crop yield could be additional (Ceglar et al. 2011). The APSIM (Agricultural production systems simulator) is a model with the potential to simulate the effect of thermo-temporal variations on crop growth, development, and physiology and provides alternative management practices for sustainable crop production (Keating et al. 2003; Holzworth et al. 2014).

Although a single model has been applied in different maize crop management, studies focusing on a broad range of sowing dates to assess temporal variation with genotypes applying multi-crop models currently are limited. Sound model calibration is the prerequisite for testing suitable management adaptation strategies for the development of sustainable productivity (Jones et al. 2003; Rahman et al. 2019). Model parameterization under local environmental conditions is a key for reliable assessment of climate change impacts and its application for decision support (He et al. 2010). Multi-model ensembles are being preferred to reduce the uncertainty due to model structure and complexities in processes (Asseng and Ewert 2013; Ewert et al. 2015; Uusitalo et al. 2015). Similarly, uncertainty in climate impact is also connected to climate models due to the complexities in atmosphere modeling, downscaling methods, and inadequate understanding of the processes (Wilby et al. 2004; Challinor et al. 2013; Osborne et al. 2013; Rahman et al. 2018). Dynamic process-based crop models have the potential to simulate the climate change impact (Ewert et al. 2015), but model calibration should be robust to minimize the risk of error and uncertainty (Bassu et al. 2014; Uusitalo et al. 2015; Rahman et al. 2019). Multi-climate and crop models provide more accurate and reliable results than the single modeling approach (Ruane et al. 2013; Martre et al. 2015; Rahman et al. 2018). This study aims to determine climate change impact uncertainty assessment on maize production using crop and climate model combinations for a mid-century under arid to semi-arid environmental conditions. Specifically, the objectives of the study are to (1) calibrate and evaluate the CERES-Maize, CSM-IXIM-Maize, and APSIM maize crop models for simulation of crop behavior to a broad range of sowing dates with different hybrids, (2) explore the potential impact of climate change on maize productivity based on a combination of the output of five climate models with the crop models, and (3) evaluation of uncertainty in the model outputs and development of adaptations for sustainable maize production in changing climatic scenarios.

## Materials and methods

### Experimental details and environmental conditions of the study site

**Fig. 1:**
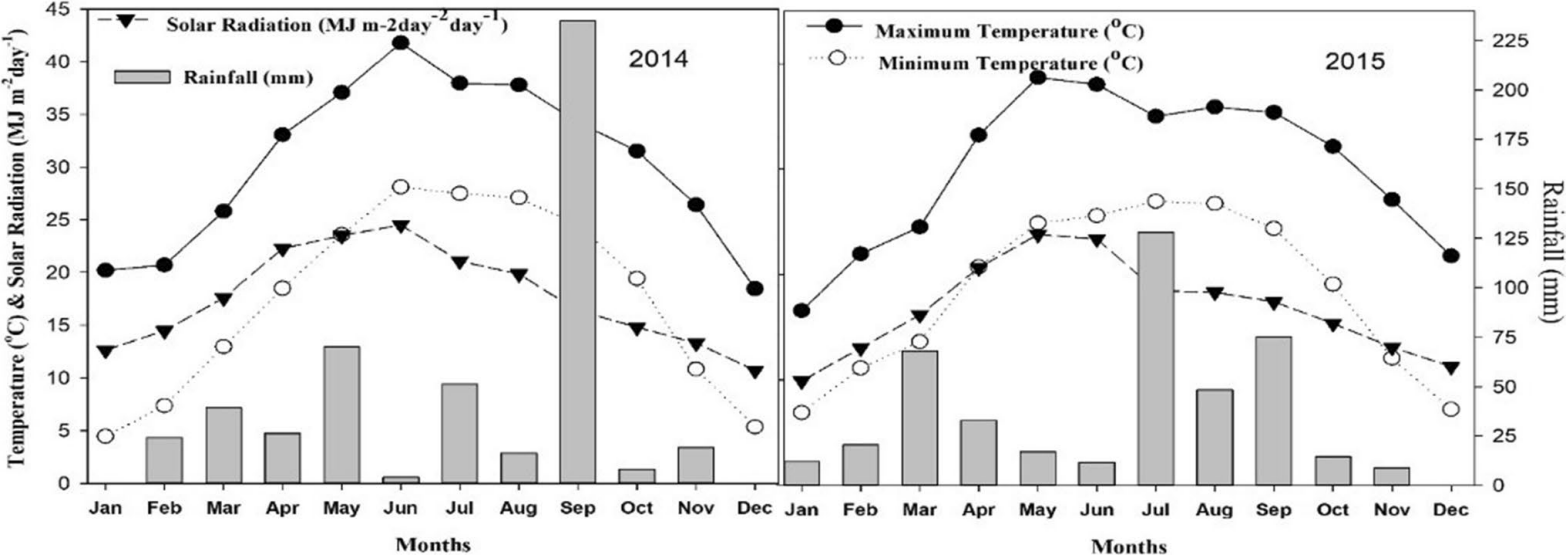
Climatic data for the spring maize growing experimental years (2014 and 2015), monthly minimum (°C), maximum (°C) temperature (°C), monthly rainfall (mm), and cumulative monthly solar radiations (M J m^−2^)

The field experiments were conducted in semi-arid region (31°30 N, 73°26 E). Mixed cropping is dominant in this region, but still, maize crop has a significant contribution in terms of the area due to industrial demand and farmer’s preference to grow maize for grain production and economic returns. The study experiences prominent changes in air temperature of day and night and even significant fluctuations during maize growing seasons. Rainfall during this season is also highly variable. Historic time series of climate observations include daily minimum (*T*min), maximum (*T*max), and precipitation, solar radiation, humidity, and wind speed were recorded by the Pakistan Meteorology Department (PMD). Detailed weather conditions during both maize growing years 2014 and 2015 can be found in the paper by Yasin et al. (2019). The soil is medium-textured with alkaline properties. The pH increases with depth. The topsoil nitrogen content is 0.06%, which decreased in the subsoil. Soil is classified as brown silty loam, and it is well-drained and calcareous. It has a very low organic carbon (OC) concentration. Detailed methodology and results are presented in a paper published by the author (Yasin et al. 2019). Soil hydrological properties such as field capacity (drained upper limit = DUL), permanent wilting point (lower limit = LL), saturated hydraulic conductivity (SSKS), and saturated soil water content (SSAT) were presented in Table 1. The four well-adopted maize hybrids (DK-6103, NK-8711, P-30-15-43, and YH-1898) in this region were sown at various sowing dates having 15 days’ intervals starting from 15 January and ending on 1 June. Fifteen plants from each experimental unit were tagged randomly to monitor the data of crop developmental phases. Crop growth and physiology were observed at regular intervals and yield, and yield attributes were recorded at final harvest by following standard procedures and protocols. The experimental site experiences enormous variations in maximum and minimum temperature during the crop season. In 2014, maximum and minimum temperature was 41.8 °C and 4.5 °C, while in 2015 maximum and minimum temperature was 38.7 °C and 6.9 °C respectively. The temperature was a bit low in the early months of the sowing period, increased up to maximum in June, and then started declining to the end of the year. Rainfall variability occurs during 2015 as there were plenty of rainfalls with high intensity as compared to 2014. The monthly average values of minimum (*T*min), maximum (*T*max) temperatures, precipitation, and solar radiations for the period of study were presented in Fig. 1.

**Table 1:** Soil physical, chemical composition, and hydrological properties used for model simulations

Depth (cm)	SLCL (%)	SLOC (%)	SLSI (%)	SLHW	LL (cm cm^−1^)	DUL (cm cm^−1^)	SSAT (cm cm^−1^)	SBDM (g cm^−3^)	SLNI (%)	SSKS (cm h^−1^)	SRGF
0-15	10	0.53	56	8.3	0.090	0.253	0.505	1.23	0.04	0.68	1
15-30	13	0.20	53	8.4	0.096	0.247	0.483	1.30	0.02	0.68	1
30-45	17	0.13	53	8.2	0.115	0.266	0.479	1.31	0.02	0.67	0.49
45-60	17	0.13	53	8.2	0.115	0.266	0.479	1.31	0.02	0.67	0.33
60-90	16	0.12	54	8.3	0.109	0.261	0.483	1.30	0.01	0.66	0.21
90-125	8	0.12	58	8.4	0.069	0.225	0.505	1.24	0.01	0.68	0.09

*SLCL* clay contents in soil, *SLOC* soil organic carbon, *SLSI* silt contents in soil, *SLHW* Soil pH in water, *LL* lower limit, *DUL* drained upper limit, *SSAT* saturation, *SBDM* soil bulk density, *SLNI* soil total nitrogen concentration, *SSKS* saturated hydraulic conductivity, *SRGF* soil root growth factor

### Data generation of climate change scenarios and selection of GCMs

Historic daily weather data of 35 years including all-weather variables (solar radiation, maximum and minimum temperature, precipitation, surface wind, dew point temperature, relative humidity, and vapor pressure) were named as baseline data (1980–2015). This baseline data set was tested for quality by following the standard protocols, and then the data set was used for future climate scenario generation using the output of 29 GCMs from the Coupled Model Inter-comparison Project (CMIP5) (Ruane et al. 2015). Depending on how baseline climatic data with daily changes in weather variables was executed, the further detailed methodology can be found in Rahman et al. (2018) and Ahmad et al. (2018). Further, mean and variability change scenarios were also deployed using stretched distribution approach that is related to quantile mapping (Ruane et al. 2015) for all GCMs for calibration. Then, climate change scenarios for the study region were developed for all GCMs for the period of mid-century (2040–2069) under the representative concentration pathway 8.5 (AgMIP 2013a; 2014; Ahmad et al. 2015). Further details about the methodology can be found in Rahman et al. (2018). Five less uncertain GCMs for temperature and precipitation during the maize growing season were selected out of 29 GCMs, based on maximum consensus. The percentage precipitation change vs. mean temperature change in the scenarios were used as criteria to find out the less uncertain group of GCMs for this region (Ruane et al. 2017). Further, these GCMs were classified into the following groups: HotWet (IPSL-CM5A-MR), HotDry (CMCC-CMS), Middle (NorESM1-M), CoolWet (CESM1-BGC), and CoolDry (INMCM4). The Several GCMs depend on deviation (ensemble standard) in the temperature and rainfall variations during maize growing season. CO_2_ concentrations of 380 ppm and 571 ppm were used in this study for baseline (Rosenzweig et al. 2013) and mid-century conditions (Taylor et al. 2012), respectively under RCP 8.5.

### Description of crop models

The CSM-CERES-Maize, CSM-IXIM, and APSIM-Maize models were tested in this study. These three models were selected based on their various in-built characters mentioned below and to evaluate their performance in local climatic conditions. The CSM-CERES-Maize and CSM-IXIM models under the DSSAT version 4.7.5.0 were used in this study (Hoogenboom et al. 2019). These models simulate the phenology, growth, and yield of maize while having an interactive effect on plant genetics, soil characteristics, crop management, and environmental conditions (Jones et al. 2003; Lizaso et al. 2013). The temperature has strong effects on growth and development phases, whereas CO_2_ affects both daily photosynthesis and transpiration; a detailed description of the original CERES-Maize model is presented in Ritchie and Alagarswamy (2003). The CSM-IXIM is a newly developed maize simulation model with more mechanistic features. It was modified with a more number of improvements and new modules from the CSM-CERES that contain leaf area expansion and senescence. The CSM-IXIM has better potential to simulate the LAI, cob growth, grain number, grain yield, carbon assimilation, partitioning, and nitrogen accretion and distribution. Two more genetic coefficients for the simulation of per-leaf foliar surface estimated LAI more accurately considered in the CSM-IXIM than CSM-CERES. These are simulated using sigmoidal functions to elucidate the expansion, longevity, and senescence of individual leaves (Lizaso et al. 2011, 2018; Yakoub et al. 2017). The combination of approaches in these two models CM-KEN and CM-SAT was used to develop the APSIM-Maize model (Keating et al. 2003), and it is being used for various maize cultivars around the world, under different management and climatic conditions (Bassu et al. 2014). The APSIM comprises the basic applications, cropping systems, crop management, intercropping and species interactions with water balance, soil impacts, land use studies, and crop adaptation (Keating et al. 2003). The APSIM-Maize model version 7.9 was used in this study, and further basic information about principles can be found in Keating et al. (2003) and Holzworth et al. (2014). The APSIM model can simulate soil water, C, N, and P dynamics and their interactions within the crop and its management systems by utilizing daily weather data (solar radiation, maximum and minimum temperatures, precipitation). Daily potential above-ground biomass production of various crops can be calculated using stage-related radiation-use efficiency (RUE) constrained by climate, soil water and nitrogen concentration, and available leaf area (Keating et al. 2003).

### Crop model calibration and evaluation with field data

The process of calibration reduces the difference between observed and corresponding simulated data mostly by modifying and adjusting the cultivar parameters of the models. Hybrids were calibrated and evaluated by using comprehensive field measurements about phenology, morphology physiology, growth, yield, and yield components collected in the maize growing seasons of 2014 and 2015. The sowing date (30 January 2014 for spring) faced minimum weather stress and produced maximum yield as compared with the rest of all sowing dates and was therefore used for calibration of the models for all hybrids. Details about crop management and data are presented in Yasin et al. (2019).

The genetic coefficients for the studied maize hybrids under DSSAT model for both the CSM-CERES-Maize and CSM-IXIM maize models were estimated by following the protocols of the generalized likelihood uncertainty estimation (GLUE) and sensitivity analysis tool associated with DSSAT (He et al. 2009; Rahman et al. 2019). The GLUE package optimizes the goodness of fit between observation and simulated parameter values (crop phenology, crop yield, and biomass, etc.). the GLUE package is built in the DSSAT. Before running the GLUE, the user needs to select some information such as crop type, a cultivar (from a cultivar list that is available in the DSSAT database for different crops), and the experimental treatments regarding cultivars grown in the field experiment. The GLUE runs 10,000 times to obtain the best cultivar coefficients. If these coefficients do not lead to an adequate fit between simulated and observed values, then a trial-and-error method (Mavromatis et al. 2001) was used to get a close match of observed with simulated values by varying thermal time and photothermal time requirements for different phenological phases of the hybrids. Simulations were stopped when they fulfilled the statistical requirement between observed and simulated data (Hunt and Boote 1998). Cultivar coefficients were determined successively starting from P1, P2, P5, and PHINT followed by G2 and G3 in the case of CSM-CERES-Maize and CSM-IXIM-Maize models.

The APSIM-Maize model was supplied with local input parameters which were directly recorded during field experimentation such as weather parameters, soil physical and chemical characteristics, crop management practices. Some other parameters which cannot be measured directly or whose values possess greater uncertainty (such as coefficients of crop hybrids) needed to be re-adjusted or calibrated. In this case, the model calibration was done with the data set of the field experiment in 2014 and the sowing date (30th January 2014) where the crop showed the best performance under maximum input conditions. Simulated outputs for crop growth, development, and production were then compared with observed values from the experiment. Parameters were readjusted within reasonable limits when inconsistencies between observed and simulated values were found, and the process was repeated until adequate behavior of the model was attained (Gaydon et al. 2017). Cultivar coefficients were determined successively such as < tt_emerg_to_endjuv >, < tt_flower_to_maturity>, < tt_flag_to_flower>, < tt_flower_to_start_grain,<head_grain_no_max_ub>, <x_stem_wt>in case of APSIM-Maize model.

## Model statistics

In this study, the model was evaluated using the coefficient of determination (*R*^2^), *d*-index value (Willmott et al. 1985), mean percentage difference (MPD), and root mean square error (RMSE) (Wallach and Goffinet 1989) between simulated and observed data, while models were compared with the formula equation of model efficiency (EF). The *d*-index value was calculated using the following equation:

1 $$d=1-\left[\frac{\sum_{i=1}^n\left(P_i-O_i\right)^2}{\sum_{i=1}^n\left(\left|P_i'\right|+\left|O_i'\right|\right)^2}\right],0\leq d\leq1$$

where, *n* = number of observations, *P*_*i*_ = predicted value for the *i*th measurement, *O*_*i*_ = observed value for the *i*th measurement, $\overline{O }$ = the overall mean of observed values, ${P}_{i}^{^{\prime}}={P}_{i}-\overline{O }$ and ${O}_{i}^{^{\prime}}={O}_{i}-\overline{O }$. The RMSE is the root mean square error, which was calculated using the following equation:

2 $$RMSE=\sqrt{\frac{{\sum }_{i=1}^{n}{{(P}_{i}-{O}_{i})}^{2}}{n}}$$

where *n* denotes the number of the observations used for comparisons, ${P}_{i}$ are the simulated values while ${O}_{i}$ are the observed ones used in the above equation. RMSE was used to determine the statistical differences between observed and simulated variables; it could be computed by using Eq. 2 to determine the predictability degree (Soler et al. 2007). Higher *d*-index value and lower RMSE value indicated a good fit between the simulated and observed data. *R*^2^ (coefficient of determination) and *d*-index values range from 0 to 1, and perfect agreement between observed and simulated data is represented by closer to 1.

Mean percent deviation indicates the deviation of simulated from observed values, a negative change revealed the underprediction while a positive change indicates the overprediction of model values.

3 $$EF=1-\left[\frac{\sum_{{\varvec{i}}}{\left({{\varvec{O}}}_{{\varvec{i}} }- {{\varvec{P}}}_{{\varvec{i}}}\right)}^{2}}{\sum_{{\varvec{i}} }{\left({{\varvec{O}}}_{{\varvec{i}}}-\overline{{\varvec{O}} }\right)}^{2}}\right]$$

The efficiency of a model (EF) showed the accuracy and model performance, and it can be computed by using Eq. 3, where ${O}_{i}$ is the ith observation recorded, ${{P}^{{^{\prime}}}}_{i}$ is the ith model simulation and $\overline{O }$ is the average overall observations.

### Climate change impact assessment and adaptation management development for sustainable maize production

Historical daily weather observations comprising 35 years (1980–2015) temperature, solar radiation, rainfall, and atmospheric CO_2_ (360 ppm) were used as baseline climate data. The quality of observed weather data was checked, and data sets were converted adopting the AgMIP format (Rosenzweig et al. 2013; Ahmad et al. 2015). Climate change projections were generated using the output of the five selected GCMs from the latest CMIP5 family (Taylor et al. 2012) under RCP 8.5 scenarios (CO_2_ concentration at 571 ppm). Calibration treatment with all its management (sowing, fertilizer, and irrigation) is used for the climate change impact assessment. The five GCMs were selected to represent the uncertainty in projected temperature and rainfall changes based on five possible climate characteristics (cool/wet, cool/dry, hot/wet, hot/dry, middle). These monthly changes were then imposed on the baseline climate series for the two seasons used in the analyses using a distribution approach (Ruane et al. 2014). Crop management options like sowing time, planting density, nitrogen application, irrigation strategies, soil fertility potential, and crop genetic potential (duration, thermal time, and genetic makeup for yield) were tested in the models to find the best management practices for sustainable maize production in the future (Rahman et al. 2018). Maize crop models were calibrated with sowing date (30 January 2014) and then validated with other nine sowing dates. The calibrated models were run across seasons for the proposed adaptations in case of sowing dates, i.e., 15-day interval treatments of sowing dates were checked for early and late sowing dates for all five GCMs during mid-century RCP 8.5. Similarly, for N application and irrigation management were tested to assess the potential of hybrids under future climate to develop the adaption for sustainable maize production.

## Results

### Historic weather data and future climate trends

Daily weather data of the previous 35 years (1980–2015) temperature, solar radiations, rainfall, and atmospheric CO_2_ were used as baseline climatic data. A warming trend is accelerating with short winter spells and delayed monsoon season. The increase in average daily maximum temperature of up to 0.47 °C has been observed, and the incidence of heatwaves has been recorded. Average annual rainfall has been decreased by 10–15%, whereas distribution and occurrence of drought spells are uneven. Results from the climate scenarios revealed that both minimum and maximum temperature will increase, and higher variability in precipitations in the maize growing season is expected in all five GCMs with RCP 8.5 scenarios during the mid-century (2040–2060). All the five GCMs predicted an increase with a range of 2.79–4.28 °C in temperature on an average during mid-century, while for rainfall there might be a change of 6–248 (mm) during the mid-century period.

### Calibration of maize models

Introducing or adding any new cultivars/hybrids into the crop models, many of the genetic parameters had to be adjusted according to their local condition, and this process is known as calibration. Four maize hybrids (DK-6103, NK-8711, P-1543, and FH-1898) were calibrated separately with the sowing date (30 January) for the year 2014. Six crop parameters (days to anthesis, days to maturity, maximum leaf area index, total dry matter, grain yield, and harvest index) observed during field experimentation were used in the calibration process. The genetic coefficients of the three models were readjusted to match the simulated and observed values of days to anthesis and maturity, grain yield, biomass at harvest, harvest index, and maximum LAI. The CSM-CERES-Maize requires a set of six cultivar genetic coefficients for simulation of phenology, growth, and grain yield, whereas CSM-IXIM-Maize has eight and APSIM 14 cultivar-specific genetic coefficients (Table 2). The calibrated values of the genetic coefficients of the three models for four spring maize hybrids (DK-6103, NK-8711, P-1543, and FH-1898) are presented in Tables 2 and 3.

**Table 2:** Genetic coefficients of spring maize hybrids adjusted in the CSM-CERES-Maize and IXIM-Maize models during calibration

Model	Cultivar coefficients	Calibrated value			
		DK-6103	NK-8711	P-1543	FH-1898
CERES-Maize	P1 (°C d)	282.0	276.0	276.0	285.0
	P2 (d)	0.660	0.580	0.580	0.620
	P5 (°C d)	795.0	857.0	860.0	870.0
	G2	817.0	672.0	660.0	795.0
	G5 (mg d^−1^)	10.90	10.30	9.90	9.12
	PHINT (°C d)	38.90	39.40	38.90	40.0
IXIM-Maize	P1 (°C d)	255.0	250.0	260.0	275.0
	P2 (d)	0.930	0.810	0.900	0.760
	P5 (°C d)	800.0	770.0	700.0	855.0
	G2	750.0	751.0	772.0	690.0
	G5 (mg d^−1^)	7.90	8.40	7.95	7.80
	PHINT (°C d)	41.00	40.00	39.00	40.00
	AX (cm^2^/leaf)	760.0	780.0	760.0	750.0
	ALL (°C d)	850.0	866.0	800.0	790.0

**Table 3:** Genetic coefficients of spring maize hybrids adjusted in the APSIM-Maize model during calibration

Model	Cultivar coefficients	Calibrated value			
		DK-6103	NK-8711	P-1543	FH-1898
APSIM-Maize	tt_emerg_to_endjuv	160.5	160.5	171.2	165.4
	tt_flower_to_maturity	650	636	643	640
	head_grain_no_max	481.2	524.6	490.3	512.2
	grain_gth_rate	13.00	13.00	12.50	13.60
	x_stem_wt units="g/stem"	200	250	230	228
	y_height units="mm"	2900	2910	2775	2800
	est_days_endjuv_to_init	1	1	1	1
	tt_endjuv_to_init	1.8	1.7	1.5	1.8
	photoperiod_crit1	10.6	11.5	10.8	10.6
	photoperiod_crit2	24.0	25.0	22.0	24.0
	photoperiod_slope	7.9	7.5	7.7	7.7
	tt_flag_to_flower	34.0	35.0	36.4	35
	tt_flower_to_start_grain	114	115	114	116
	tt_maturity_to_ripe	0.1	0.0	0.1	0.1

### Evaluation of model performance with respect to mean percentage difference

#### Model response to duration of major phenological events

The results showed significant differences among all three crop models (*P*<0.01) in terms of mean percent difference between observed and simulated values for days to anthesis and days to maturity (Table 4). The CSM-IXIM-Maize model showed a higher mean percent difference between observed and simulated for hybrid DK-6103 and NK-8711 when compared with the CERES-Maize and APSIM-Maize model as both exhibited lesser mean percent difference in case of days to anthesis and days to maturity. Root mean square error was higher for simulations of CSM-IXIM-Maize model (Table 3). Mean percent difference between observed and simulated days to anthesis and days to maturity for hybrid P-1543 was found (−1.47 and −0.70) respectively in the APSIM-Maize model, due to higher differences in a simulation of days to anthesis and maturity than observed values. The CERES-Maize model predicted high (−3.57 and −2.56) mean percent difference in case of days to anthesis and days to maturity respectively. Mean percent difference between observed and simulated days to anthesis for hybrid FH-1898 was found non-significant for all three models, whereas the CSM-IXIM-Maize model gave more (0.193) mean percent difference in case of days to maturity. Root mean square error was recorded higher in simulations of the APSIM-Maize model (Table 4).

**Table 4:** Comparison of mean percent difference (%) of model simulations for phenological, growth, and yield parameters during calibration of spring hybrids (different letters denote significant differences between models at *P*<0.05) (*N*=9)

Hybrids	Treatments Crop models	MPD (%)					
		Days to anthesis	Days to maturity	LAI	TDM (kg ha^−1^)	Yield (kg ha^−1^)	HI
DK-6103							
	CERES-Maize	2.72 B	1.13 B	12.28 B	1.31 A	3.28 A	22.65A
	CSM-IXIM-Maize	5.53 A	3.45 A	12.62 B	−1.32 B	2.27 C	13.53 B
	APSIM-Maize	1.21 C	0.76 C	20.29 A	−2.45 C	2.44 B	2.44 C
	HSD (0.05)	0.06	0.03	3.08	0.043	0.029	0.03
	Significance	**	**	**	**	**	**
NK-8711	CERES-Maize	−3.62 B	−2.78 B	6.6917 B	−1.17 A	3.37 B	13.57 A
	CSM-IXIM-Maize	−4.48 C	−2.77 B	1.4575 C	−4.54 B	0.50 C	11.32 B
	APSIM-Maize	−0.32 A	−0.19 A	12.698 A	−5.04 C	9.10 A	9.10 C
	HSD (0.05)	0.076	0.031	0.526	0.043	0.039	0.038
	Significance	**	**	**	**	**	**
P-1543	CERES-Maize	−3.57 C	−2.56 C	17.88 A	−0.28 A	1.01 B	18.29 A
	CSM-IXIM-Maize	−2.68 B	−1.45 B	14.71 B	−1.36 C	1.34 A	14.96 B
	APSIM-Maize	−1.47 A	−0.70 A	14.22 B	−1.10 B	−0.04 C	−4.04 C
	HSD (0.05)	0.70	0.50	0.526	0.044	0.0384	0.0381
	Significance	**	**	**	**	**	**
FH-1898	CERES-Maize	0.109	−0.37 B	8.30 B	−1.90 C	−0.84 C	6.07 B
	CSM-IXIM-Maize	0.106	0.193 A	8.30 B	−0.53 B	−0.14 A	11.8 A
	APSIM-Maize	−0.20	−0.94 C	9.56 A	−0.30 A	−0.38 B	−0.38 C
	HSD (0.05)	0.399	0.419	0.525	0.043	0.039	0.037
	Significance	NS	**	**	**	**	**

#### Crop model response to maize growth (leaf area index and biomass)

Significant differences were found among all three crop models (*P*<0.01) in terms of the mean percent difference between observed and simulated values of leaf area index, total dry matter (Table 4). The APSIM-Maize model predicted greater values of the mean percent difference between observed and simulated values of maximum leaf area index for hybrid DK-6103, NK-8711, and FH-1898, while the other two models were statistically at par with each other for mean percent difference of leaf area index. The CERES-Maize model gave a higher mean percent difference in case of total dry matter due to higher simulation of total dry matter than observed total dry matter. The APSIM-Maize model presented a high simulation of total dry matter then observed total dry matter with the mean percent difference of −2.45. The MPD between observed and simulated for hybrid P-1543 were recorded higher (17.68) in CERES-Maize model for maximum leaf area index. All models showed oversimulation trend for total dry matter. Root mean square error was greater in simulations of the CSM-IXIM-Maize model (Table 4).

#### Model performance and response to yield and yield components of different hybrids

The results revealed significant differences among all three crop models (*P*<0.01) in terms of the mean percent difference between observed and simulated values for grain yield and harvest index (Table 4). The CERES-Maize model predicting a higher mean percent difference between observed and simulated for hybrid DK-6103 recorded more (3.28 and 22.65) for grain yield and harvest index respectively, whereas the CSM-IXIM-Maize showed less mean percent difference for grain yield, and the APSIM-Maize model gave less mean percent difference (2.44) in the case of harvest index. The mean percent difference between observed and simulated for hybrid P-1543 and FH-1898 was found higher in the CSM-IXIM-Maize model in the case of grain yield. The APSIM-Maize model over-stimulated the grain yield of hybrid NK-8711 and depicted a higher mean percent difference between observed and predicted values. Root mean square error was higher in simulations of the CERES-Maize model and CSM-IXIM-Maize model (Table 4).

### Evaluation of model performance for the coefficient of determination, RMSE, and d-index

**Fig. 2:**
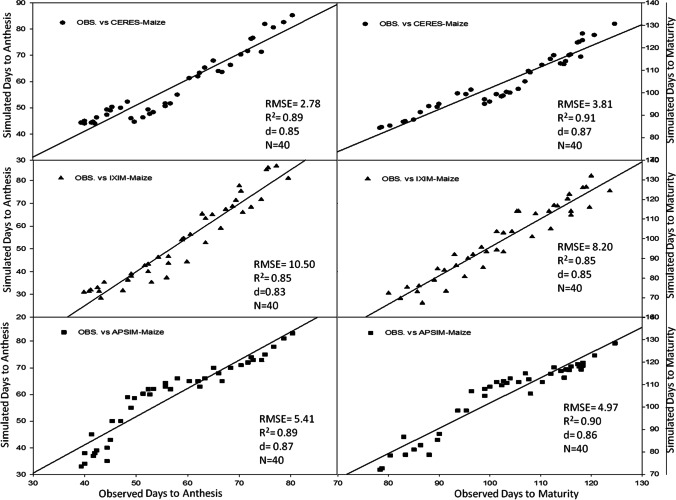
Relationship between observed and simulated values of days to anthesis and days to maturity for spring maize hybrids sown at various dates during 2014–2015

Maize multi-models were evaluated for behavior by analyzing the relationship between simulated and observed values of all output variables which were used in the process of calibration such as days to anthesis, days to maturity, leaf area index, total dry matter (kg ha^−1^), grain yield (kg ha^−1^), and harvest index. All three models (CERES-Maize, CSM-IXIM-Maize, and APSIM-Maize) presented a good and smooth relationship between observed and simulated values. CERES-Maize and APSIM-Maize models showed lower RMSE values (2.78 and 5.41), higher *d*-index (0.85 and 0.87) along with reliable *R*^2^ (0.89) for days to anthesis and days to maturity respectively as compared to CSM-IXIM-Maize, which showed a bit higher RMSE (10.50) with reliable *R*^2^ value (0.85) for all sowing dates and hybrids during the spring season (Fig. 2).

**Fig. 3:**
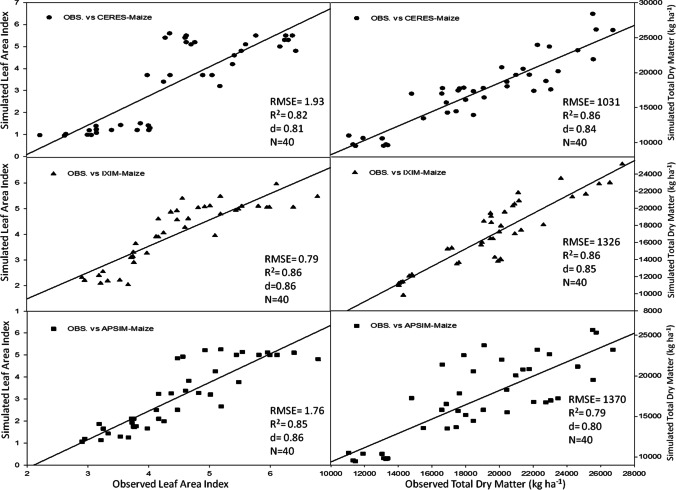
Relationship between observed and simulated values of leaf area index and total dry matter (kg ha^−1^) for spring maize hybrids sown at various dates during 2014–2015

**Fig. 4:**
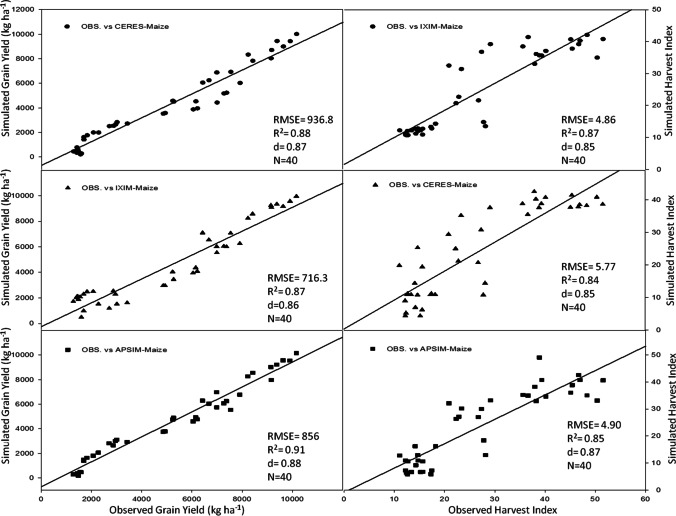
Relationship between observed and simulated values of grain yield (kg ha^−1^) and harvest index for spring maize hybrids sown at various dates during 2014–2015

The CSM-IXIM-Maize model performed well for leaf area index and total dry matter, with the lowest RMSE (0.79 and 1326), good *d*-index (0.86 and 0.85) respectively, and fair *R*^2^ value (0.86) when compared with the other two models (Fig. 3). The CSM-IXIM-Maize model performed very well for grain yield with the lowest RMSE (716.3), high d-index (0.88), and fair *R*^2^ value (0.87) when compared with the other two models, whereas for harvest index CERES-Maize predictions were better with lower RMSE (4.86), higher *d*-index (0.87), and reliable *R*^2^ value (0.85) (Fig. 4). Overall, *d*-index values were recorded higher (0.80 to 0.88) for all hybrids at all sowing dates.

### Performance of maize multi-models during the evaluation

**Fig. 5:**
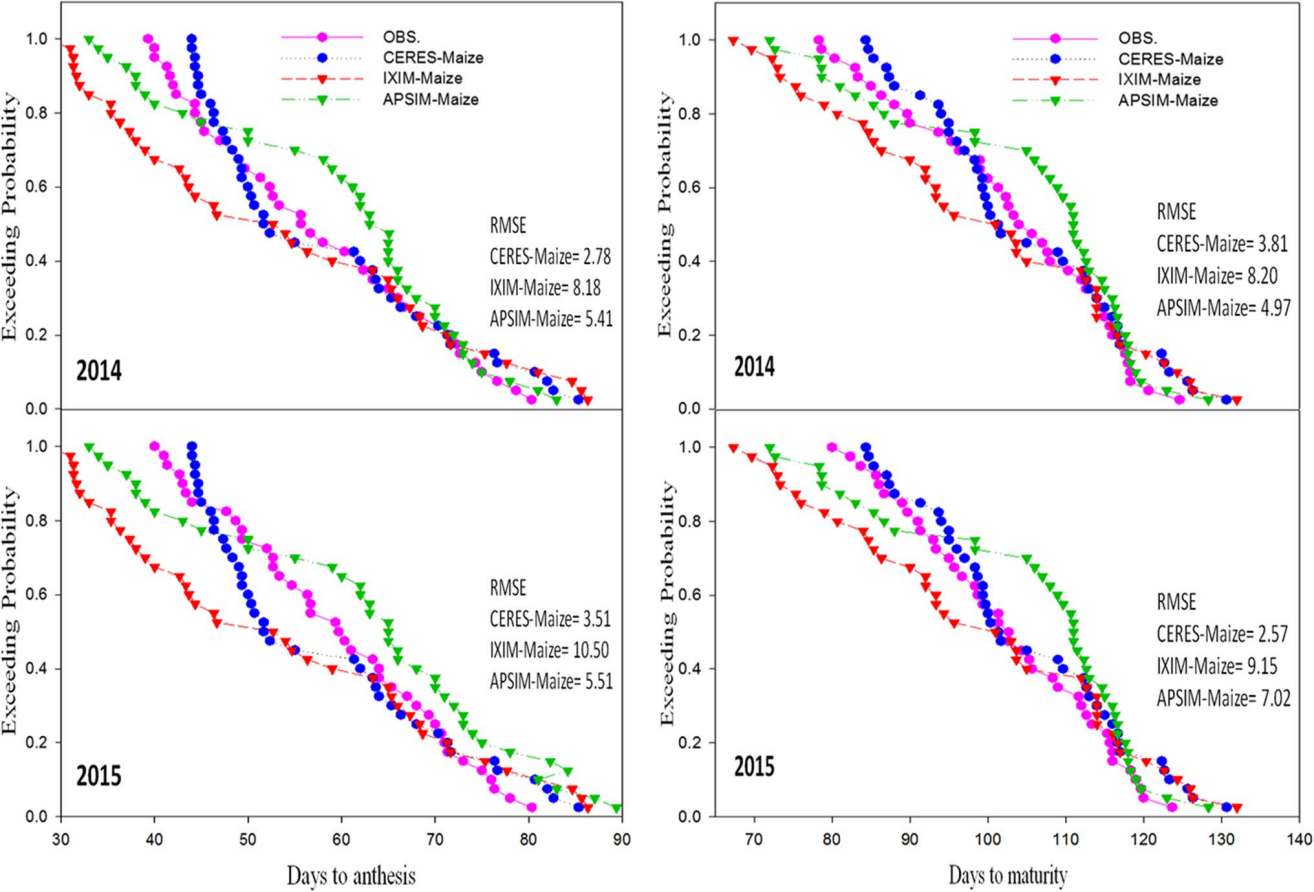
Simulating behavior of Maize multi-models for days to anthesis and days to maturity of spring hybrid maize during 2014–2015

**Fig. 6:**
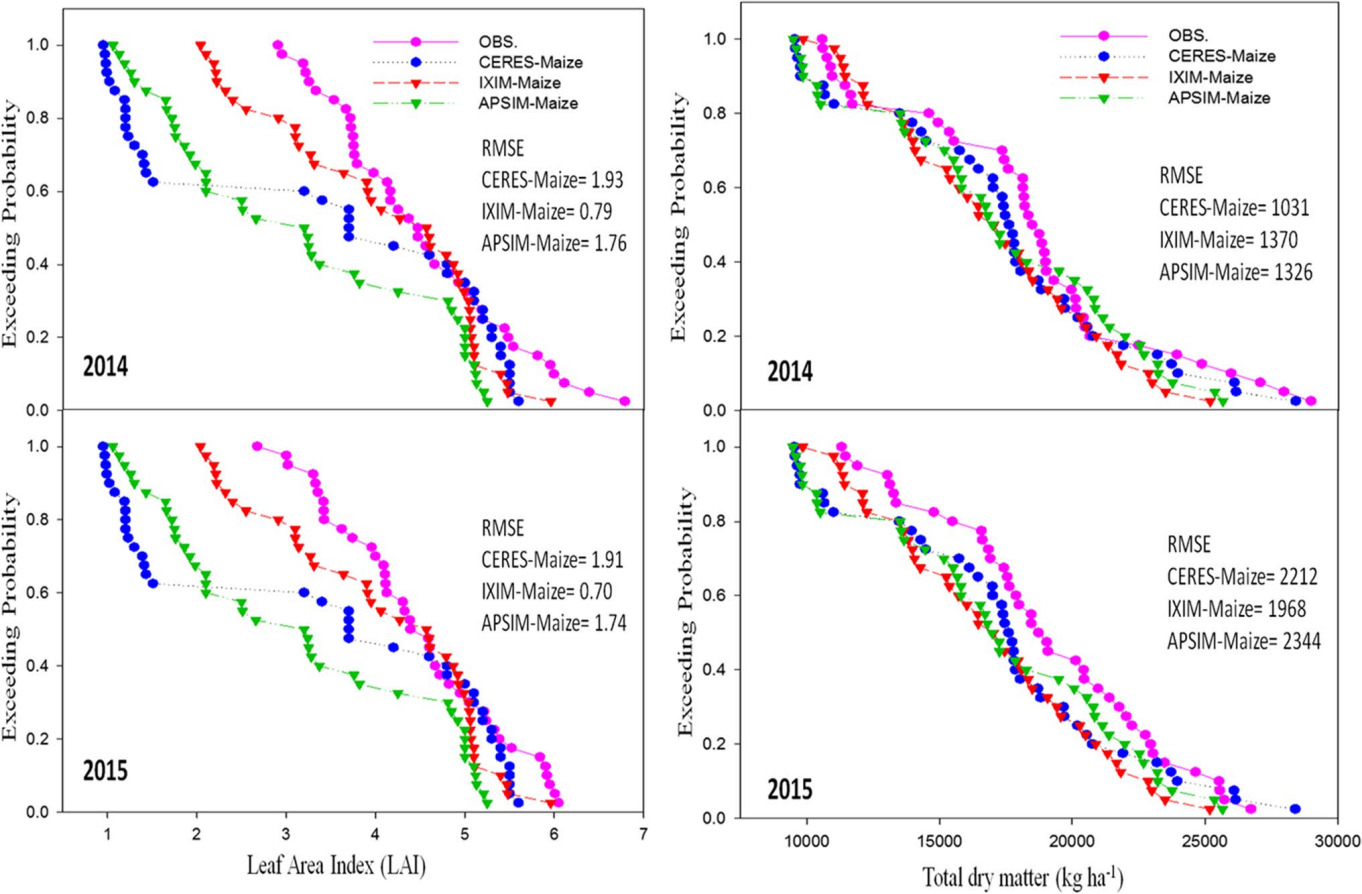
Simulating behavior of Maize multi-models for leaf area index (LAI) and total dry matter (kg ha^−1^) of spring hybrid maize during 2014–2015

**Fig. 7:**
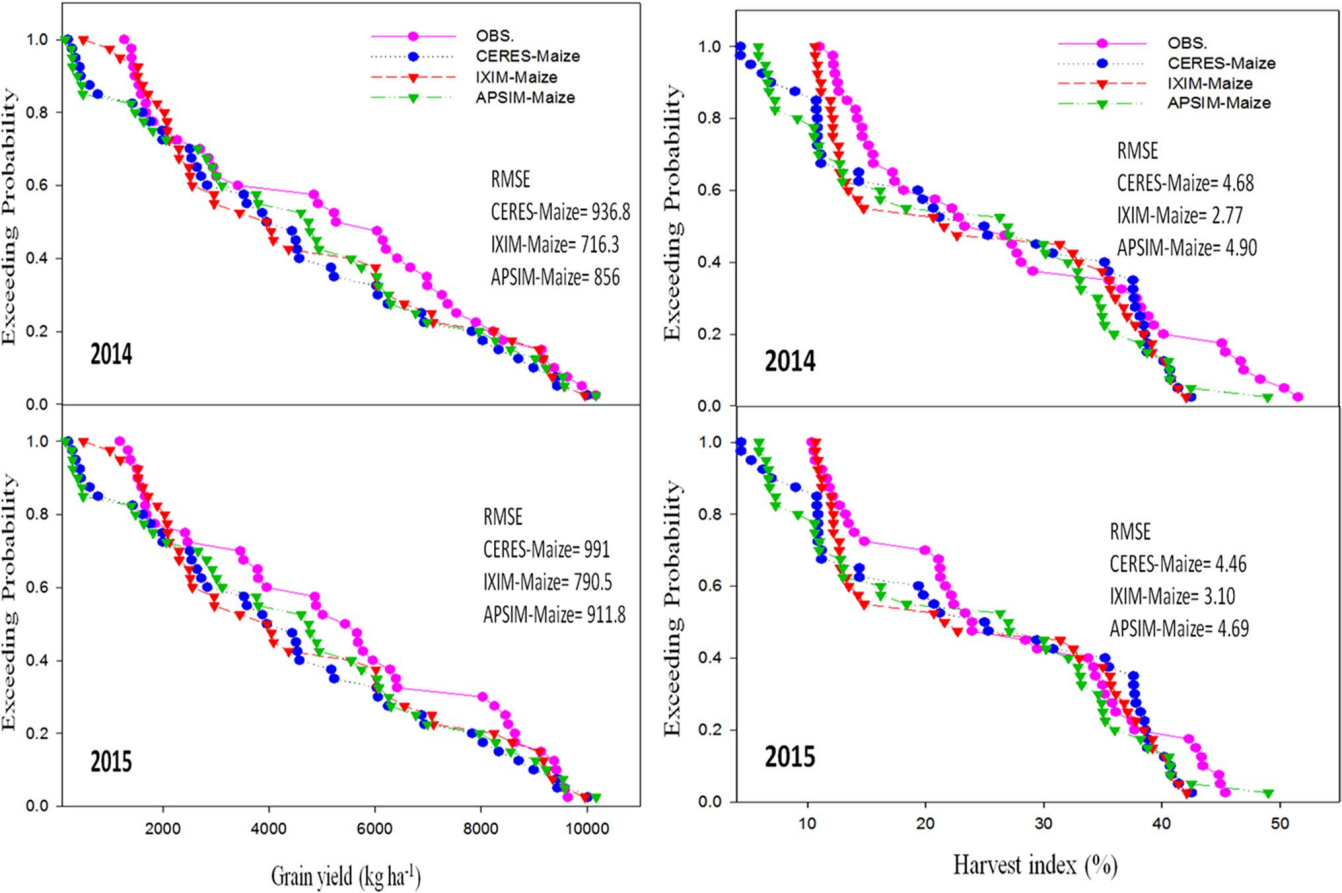
Simulating behavior of Maize multi-models for grain yield (kg ha^−1^) and harvest index of spring hybrid maize during 2014–2015

**Fig. 8:**
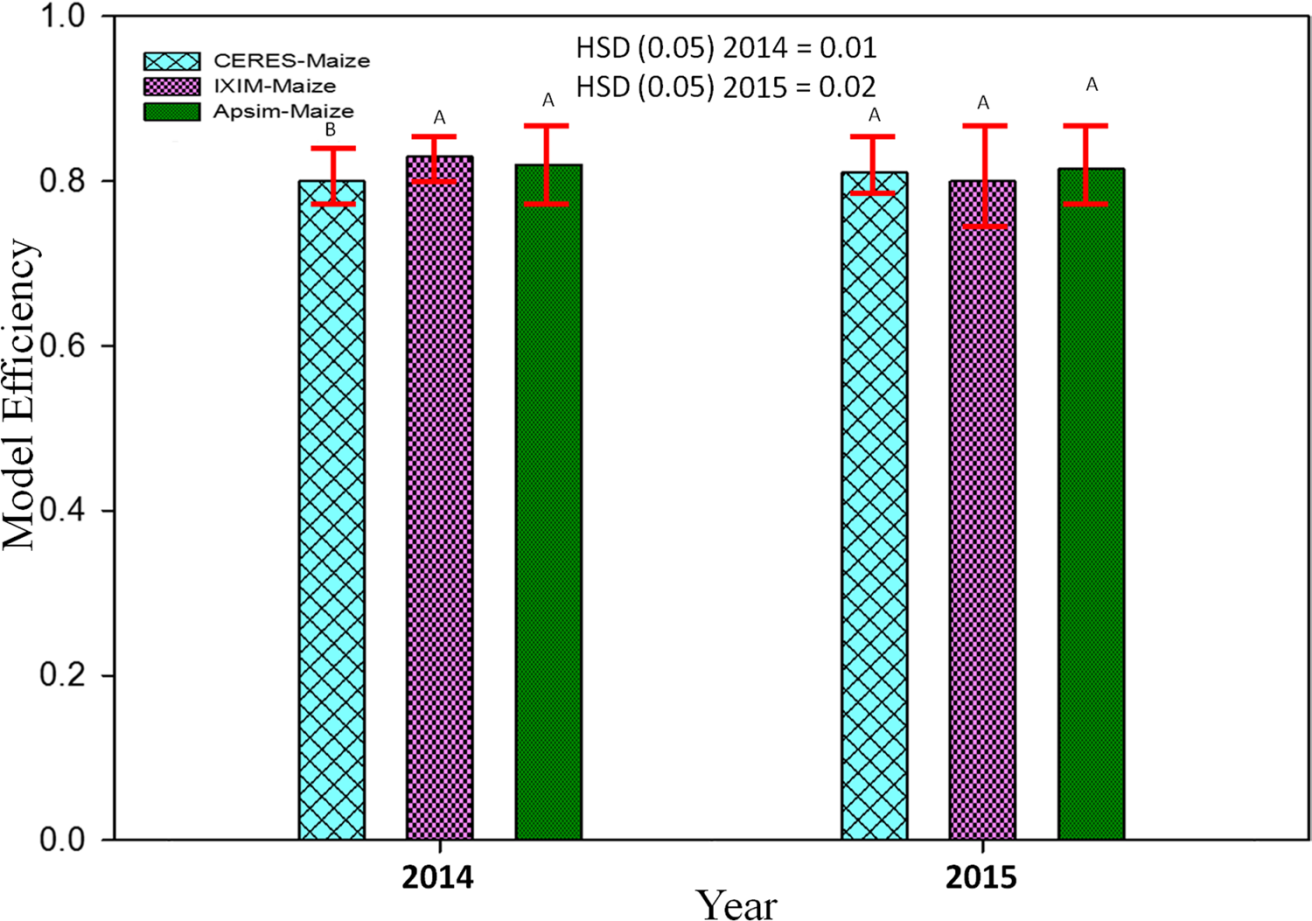
Performance efficiency of CERES, IXIM, and APSIM Maize multi-models during spring 2014–2015

Model evaluation was done for days to anthesis, days to maturity, leaf area index, total dry matter, grain yield, and harvest index to check the maize multi-model performance. Exceeding probability (Figures 5, 6, and 7) associated with the days to anthesis, days to maturity, leaf area index, total dry matter, grain yield, and harvest index during the spring season for both years (2014 and 2015). The CERES-Maize and APSIM-Maize model performance were very good for days to anthesis and days to maturity. Simulations of the CERES-Maize model were very well for days to anthesis during both years with less RMSE value 2.78 and 3.51 respectively as compared to CSM-IXIM-Maize and APSIM-Maize models (Fig. 5). A similar trend was observed for days to maturity. For leaf area index, the simulations of CSM-IXIM-Maize model were very good during both years with lower RMSE value 0.79 and 0.70 respectively as compared to other two models, whereas APSIM-Maize simulated total dry matter very well during both years with RMSE value (1326 and 1968) respectively while comparing with other models (Fig. 6). The CSM-IXIM-Maize model simulations for grain yield were good during both years with lower RMSE (716 and 790), respectively (Fig. 7), whereas the APSIM-Maize simulation for grain yield was also very good during both years with RMSE value (856 and 911.8). The harvest index was simulated very well by CSM-IXIM-Maize model during both years with lower RMSE (2.77 and 3.10), respectively (Fig. 7). The performance efficiency of all three models for crop growth, development, and yield parameters was determined during both years. Significant differences (*P*<0.05) were found among all maize models during the year 2014, whereas non-significant differences were found during 2015 (Fig. 8). The performance efficiency of APSIM-Maize and CSM-IXIM-Maize models was higher than CERES-Maize model during 2014, and these two models are statistically at par with the means of each other. On average, the simulating behavior of the CSM-IXIM-Maize model was very well under semi-arid conditions during both years (Fig. 8).

## Climate change scenarios and uncertainty assessment for climate change impacts on maize productivity

**Fig. 9:**
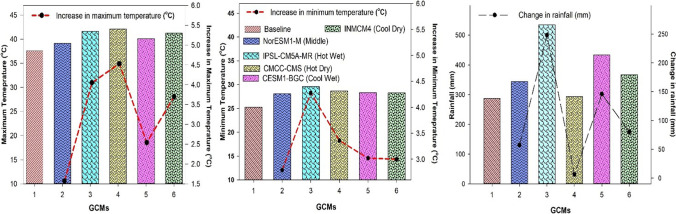
Predicted change in climatic variables by the period 2040 to 2060 for five climate models (GCMs) compared to the baseline period

A significant rise in both maximum and minimum temperature and greater variability in precipitations during maize growing season in all five GCMs with the RCP 8.5 scenarios for mid-century were observed in the climate scenarios of the five GCMs (Fig. 9). Predictions from CMCC-CMS (Hot Dry scenario) showed a higher (4.53 °C) increase in maximum temperature during mid-century, whereas NorESM1-M (Middle) GCM predicted a lower increase by 1.57 °C in the future (2040–2069) under RCP 8.5 (Fig. 9). In the case of rainfall predictions, the IPSL-CM5A-MR (Hot Wet) GCM showed an increase (248 mm) in rainfall during mid-century (2040–2069), while CMCC-CMS (Hot Dry) GCM showed a minor decrease (7 mm) in rainfall during mid-century (Fig. 9). On average, all five GCMs predicted for minimum temperature an increase of 3.27 °C and a rise of (3.29 °C) in maximum temperature during mid-century. A positive change with an increase of 107 mm was observed on an average in rainfalls during mid-century as predicted by all five climate scenarios (Fig. 9).

**Fig. 10:**
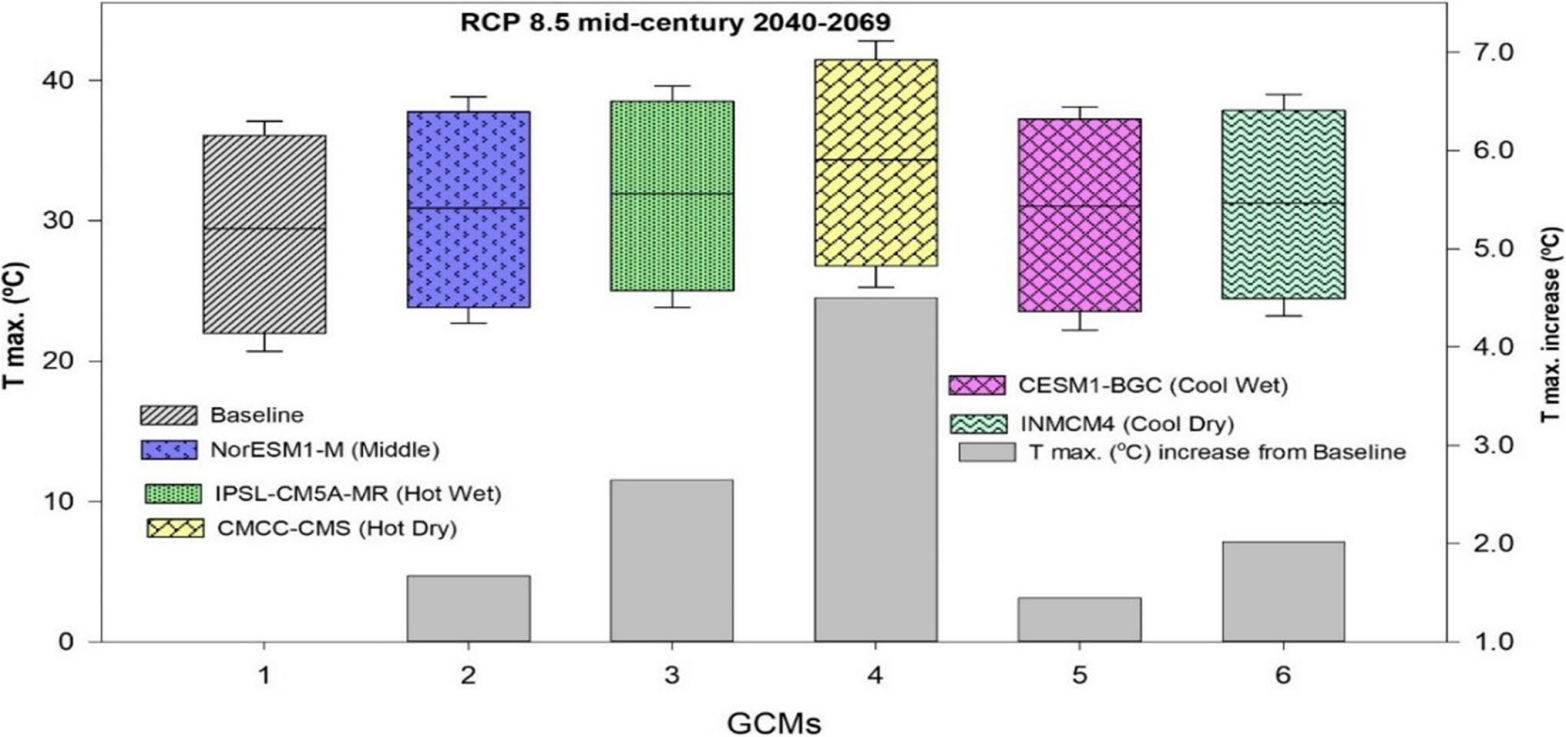
Predicted change in monthly mean maximum temperature (°C) during the spring season (February, March, April, May) in future (2040–2069) under representative concentration pathways (RCPs) 8.5 through selected GCMS. *(1=baseline, 2=NorESM1-M, 3= IPSL-CM5A-MR, 4= CMCC-CMS, 5= CESM1-BGC, and 6= INMCM4)

**Fig. 11:**
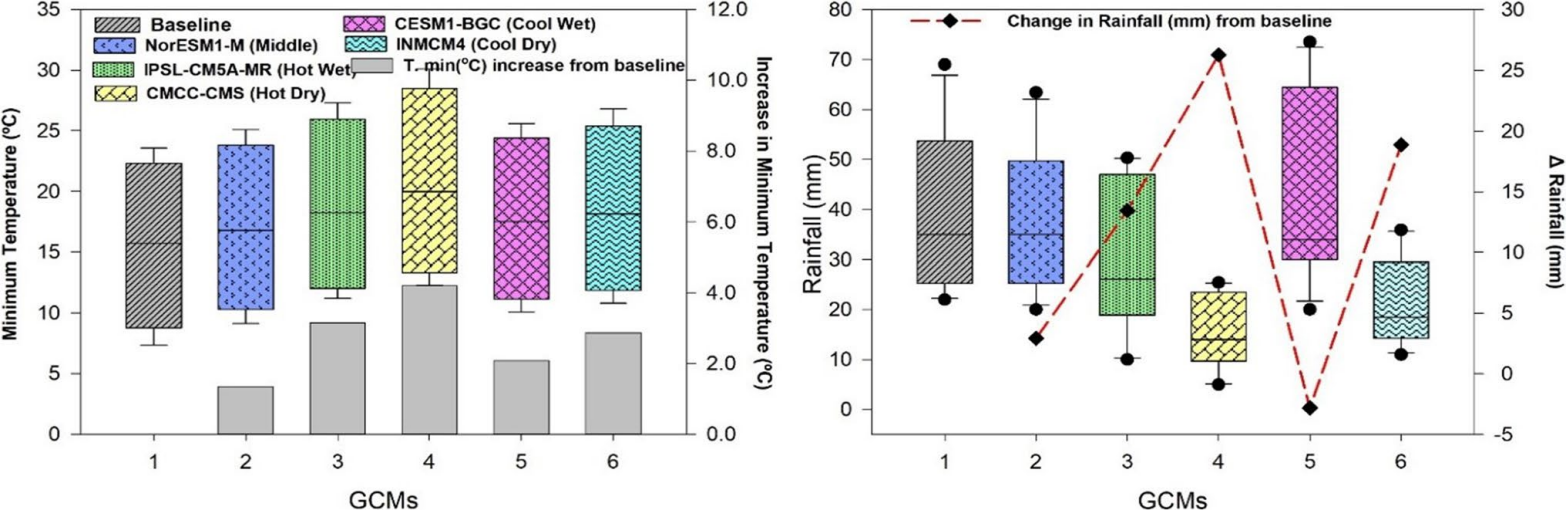
Predicted change in monthly mean minimum temperature (°C) and rainfall (mm) during the spring season (February, March, April, May) in future (2040–2069) under representative concentration pathways (RCPs) 8.5 through selected GCMs. *(1=baseline, 2=NorESM1-M, 3= IPSL-CM5A-MR, 4= CMCC-CMS, 5= CESM1-BGC, and 6= INMCM4)

**Fig. 12:**
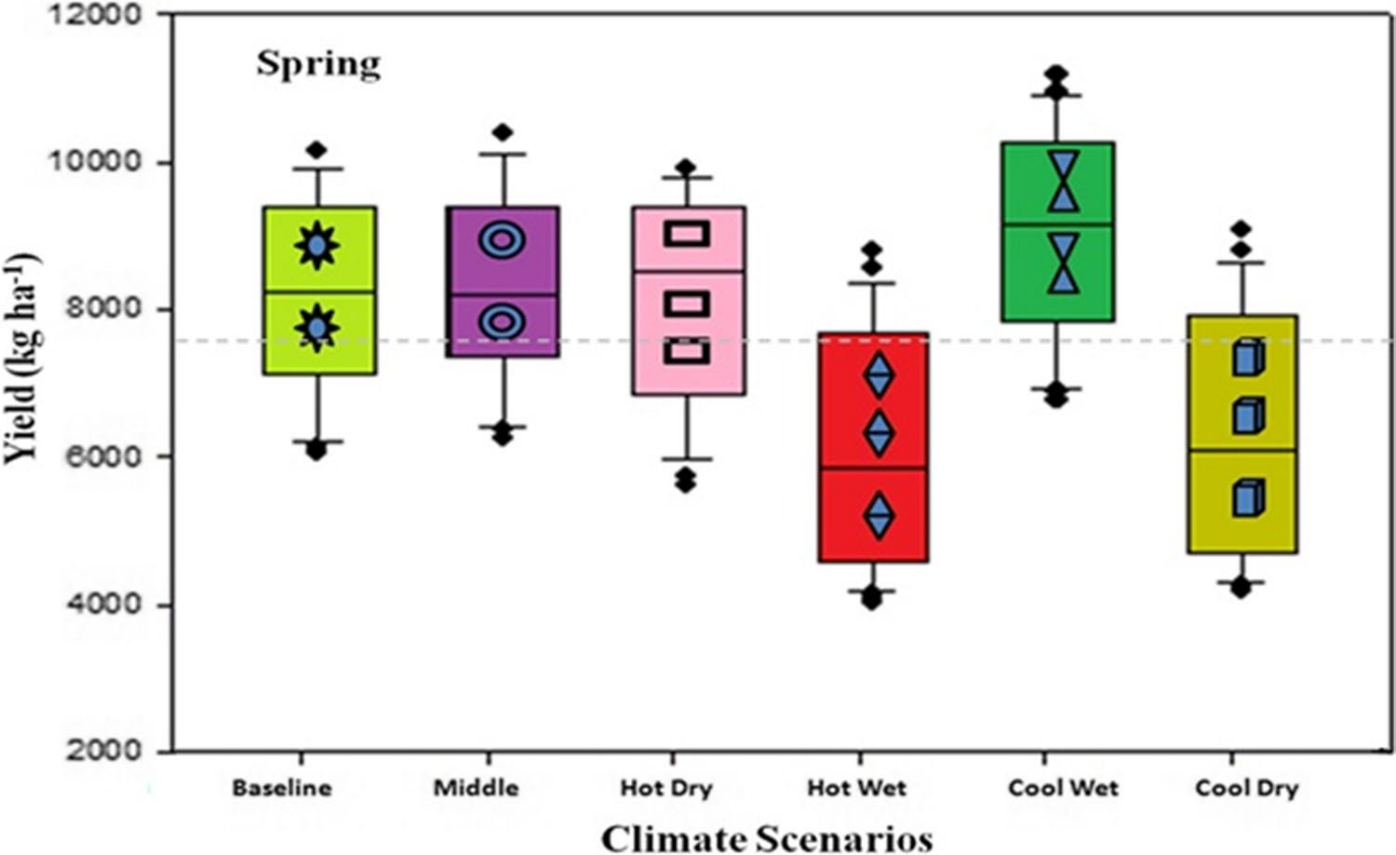
Impact of climate change on spring (February, March, April, May) maize planting under different climate change scenarios

Variation in the changes of maximum, minimum temperature, and rainfall was observed in the outputs of the five different climate models by a mid-century period during the spring season (Figs. 10 and 11). A higher increase in maximum and minimum temperature of 5.03 and 4.99 °C respectively was predicted under the “Hot Dry” climate scenario (Figs. 10 and 11). Rainfall may also vary from 15 to 43 (mm) during these months (Fig. 11) as compared to other climate models. Under Cool Wet climate scenarios during mid-century a slightly lower increase was noted in maximum and minimum temperature 1.44 and 2.07 °C respectively (Figs. 10 and 11). In the simulations by all three crop models, either positive or negative impacts on maize production were observed depending on the climate scenarios. Average maize yield is expected to reduce from 440 to 2047 kg ha^−1^ as predicted by middle and hot wet climate scenarios during the spring season, respectively (Fig. 12). Spring season maize yield may experience a decline of up to 2047 kg ha^−1^ from current yield under hot dry climate change scenario, while cool wet climate scenario predicted an increase 40–150 kg ha^−1^ in yield from baseline in mid-century (Fig. 12).

**Fig. 13:**
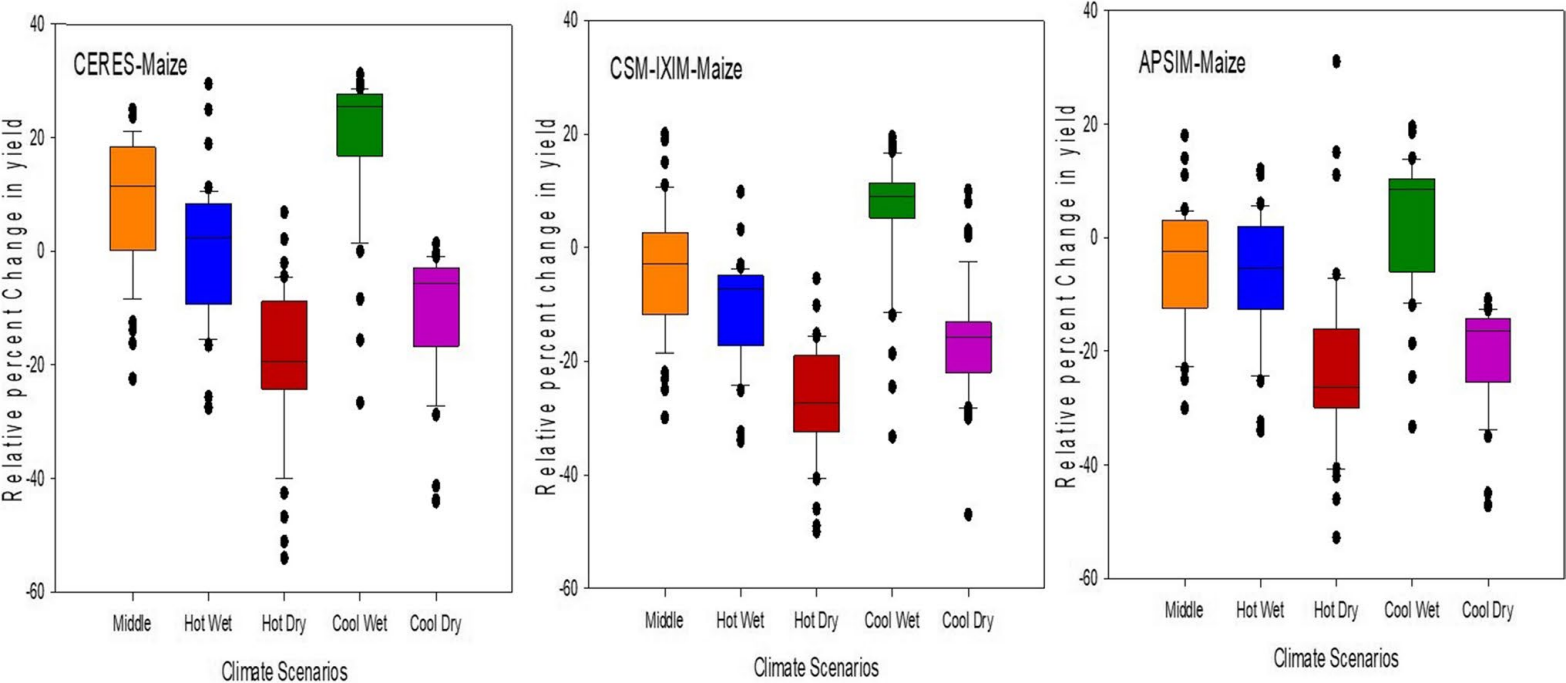
Relative change in grain yield in response to future climate (2040–2069) during spring with three crop models under five climate scenarios

Prediction of all crop models such as CERES-Maize, IXIM-Maize, and APSIM-Maize along with climate models showed a decline in yield from 8 to 55% from baseline in the spring season during mid-century, except in the “Cool Wet” scenario (Fig. 13). A very huge reduction in yield is expected up to 55%, 50%, and 52% under the “Hot Dry” scenario as predicted by CERES-Maize, IXIM-Maize, and APSIM-Maize respectively. All three models indicated an increase in yield under the “Cool Wet” scenario. The CERES-Maize model predicted an average yield increase of 6–8%, whereas IXIM and APSIM-Maize predicted an increase in yield from 8 to 19% in the future mid-century under cool wet climate scenarios (Fig. 13).

## Adaptations for spring maize crop under climate change scenarios (2040–2069)

**Fig. 14:**
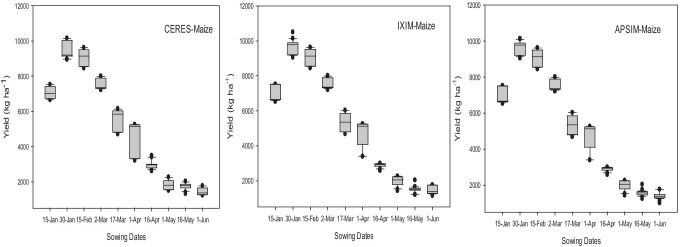
Effect of sowing dates on grain yield (kg ha^−1^) in response to future climate (2040–2069) during spring crop season with three crop models

The calibrated maize crop models were analyzed to determine the impact of adaptation (modification in sowing date, N application rates, and irrigation application). The adaptation options were selected to assess their potential to alleviate the negative impacts of climate change on maize yield with the ultimate objective to get an optimum adaptation package. Fifteen (15) days earlier than the current sowing time (mid-February) performed best in all GCMs than other planting dates (Fig. 14).

**Fig. 15:**
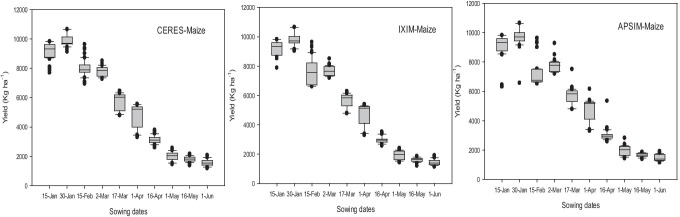
Effect of sowing dates in combination with varying fertilizer and irrigation practices on grain yield (kg ha^−1^) in response to future climate (2040–2069) during spring crop season with three crop models

Three nitrogenous fertilizer levels (10%, 20%, and 30%) increased from recommended nitrogen rate applied in three splits @ 220 kg ha^−1^ in this study were tested along with the sowing date to develop combined adaptation practice for future climate. An increase in 20% of nitrogen fertilizer application then-current rate sustained the maximum grain yield of maize when planted early in the spring season. A similar above-mentioned practice was repeated for determining the irrigation adaptation. Irrigation treatments 80% and 90% of 100% full irrigation (recommended) were tested in which 90% of irrigation was found as a good adaptation practice to get maximum yield return (Fig. 15). These practices may act as a good production package to obtain maximum maize productivity under mid-century climatic scenarios as predicted by all crop and climate models.

## Discussion

Using crop growth models for the determination of crop growth, yield, and developmental phases at different sowing dates is a very helpful technique for various climatic conditions. The researcher used modeling techniques to get assistance in developing the information regarding the short-term and long-term organization of agricultural activities (Aurbacher et al. 2013). Multi-models may have different simulating behavior under various environmental conditions such as a series of sowing times. In the current study, the performance of three maize models (CERES-Maize, IXIM-Maize, and APSIM-Maize) was tested at various sowing dates and hybrids. The genetic coefficients for DK-6103, NK-8711, P-1543, FH-1898 were well-calibrated in all crop models (Tables 1 and 2). Genetic coefficients of DSSAT modules and APSIM-model were variate as previously reported (Knorzer et al. 2011; Kassie et al. 2014). The genetic coefficient may vary among hybrids and also due to climatic conditions (Araya et al. 2015). All models simulated crop phenology, growth, and yield very well with satisfactory mean percent difference values during the process of calibration of models (Table 3). For phenological attributes, APSIM-Maize and CERES-Maize models predicted lesser mean percent difference as CERES-Maize and APSIM-Maize model showed days to anthesis and days to maturity with lower RMSE values (2.78 and 5.41), higher *d*-index (0.85 and 0.87) along with fair *R*^*2*^ (0.89 and 0.89) respectively (Fig. 2). Similar results were reported by Mubeen et al. (2016) and Ban et al. (2015) that the CERES-Maize model simulated maize phenology very well with less difference between simulated and observed values under a semi-arid environment. Chenu et al. (2009) reported that the APSIM-Maize model predicted maize phenology and yield very well with less mean percent difference during the maize growing season. The CSM-IXIM-Maize model performed well for leaf area index and total dry matter with less RMSE (0.79 and 1326), good *d*-index (0.86 and 0.85) respectively, and fair *R*^2^ value (0.86) compared to the other two models (Fig. 3). The CSM-IXIM-Maize model simulates the leaf area index of the plant very accurately as it modified from CERES-Maize model with few improvements (Ban et al. 2015). The CSM-IXIM-Maize model performed well for grain yield (kg ha^−1^), with less RMSE (716.3), high *d*-index (0.88), and acceptable *R*^2^ value (0.87) compared to the other two models, whereas CERES-Maize and APSIM-Maize showed better predictions for harvest index with lower RMSE (4.86), *d*-index (0.87), and reliable *R*^2^ value (0.85) (Fig. 4). Simulations of the CSM-IXIM-Maize are very efficient for total dry matter assimilation and partitioning and grain yield (Lizaso et al. 2011). CERES-Maize, CSM-IXIM-Maize, and the APSIM-Maize could be used under different climates for simulation studies and mounting crop management activities (Hoogenboom et al. 2011; Holzworth et al. 2014).

Simulating the behavior of maize multi-models (CERES-Maize, CSM-IXIM-Maize, and APSIM-Maize) exhibited a reliable relationship between observed and simulated values for all recorded attributes. The CERES-Maize and APSIM-Maize model predicted maize phenology such as days to anthesis and days to maturity with lower RMSE (2.78 and 5.41), higher *d*-index (0.85 and 0.87) along with fair *R*^2^ (0.89 and 0.89) respectively (Fig. 2). These results seem similar to the findings of Soler et al. (2007), Tojo et al. (2007), and Gaydon et al. (2017) which reported that CERES-Maize model and APSIM-Maize can predict days to anthesis of maize very closely under different environments. The CSM-IXIM-Maize model performed well with a close prediction for lead area index, total dry matter (kg ha^−1^), and grain yield (kg ha^−1^) (Figs. 6 and 7). The CSM-IXIM-Maize can simulate biomass and grain yield of maize accurately (Yakoub et al. 2017). The APSIM-Maize simulation for the total dry matter was good with lower RMSE values during both years (Fig. 6) which is similar to the findings of Knorzer et al. (2011).

Increasing temperature and fluctuations in precipitation and drought spells are likely to decrease crop productivity in the future (Kang et al. 2009). Global general circulation models (GCMs) are well-known for their projections regarding future climates and predicted the rise in global temperature from 1.6 to 4.6 °C in the next 50 to 100 years due to high emissions of greenhouse gases (IPCC 2007). Climate change impact assessment studies may not rely upon a single GCM. The use of multiple models may be an efficient method for climate change impact assessment and its vulnerability to crop productivity (Rosenzweig et al. 2013; Asseng and Ewert 2013). Pakistan is highly susceptible to the adverse impacts of climate change. Due to extreme climatic conditions, crop productivity was negatively affected. The projected decline in major crop yield in the future is from 0.8 to 27% from the current yield. Climate change impact assessment studies and adaptation packages are required to reduce these negative effects. Rasul and Ahmad (2012) reported that the average annual temperature has increased from 0.6 to 1 °C during the past 50 years. Ahmad et al. (2015) reported an average rise in mean annual temperature from 2 to 3 °C as compared to the baseline during the mid-century RCP8.5 scenario, and rainfall may have unexpected fluctuations either increase or decrease as compared to the current rainfall pattern. These results match with the results of the current study that there would be a rise in temperature from 1.57 to 3.29 °C and enormous fluctuations in rainfall during maize growing seasons in all five GCMs with RCP 8.5 scenarios for the mid-century (Fig. 9). Results from current climate change studies showed that there would be a temperature rise either maximum or minimum and greater variability in precipitations during maize growing season in all five GCMs for the mid-century (Fig. 9). The CMCC-CMS (Hot Dry) climate scenario and NorESM1-M (Middle) predicted a higher (4.53 °C) and 1.57 °C change/increase in maximum temperature respectively during mid-century (2040–2069) under RCP 8.5. Similar to the results reported by Iqbal and Zahid (2014), in Pakistan there would be a rise up to 4.38 °C in the future. On average, all five GCMs predicted a high increase (3.27 °C) in minimum temperature and an increase of 3.29°C in maximum temperature during mid-century (Fig. 9) that would have quite negative effects on maize productivity. Ishfaq et al. (2020) reported that maximum and minimum temperature would increase from 3.4 to 3.8 °C by mid-century under RCP 8.5. IPSL-CM5A-MR (Hot Wet) GCM predicted a very high (248 mm) fluctuation in rainfall during mid-century (2040–2069), and CESM1-BGC (Cool Wet) GCM showed a change of 146 mm in rainfall during mid-century (Fig. 9). High temperature enhances the evaporative rate from the earth’s surface due to which hot air becomes moist and more rainfall occurs as a similar phenomenon was reported by Trenberth (2011). The average change of 107 (mm) in rainfalls during mid-century (2040–2069) was predicted by five different climate scenarios (Fig. 8). Climate change impacts could reduce average maize yield up to 25% by 2050 under changing climate in the future (Ishfaq et al. 2020). The climate models along with crop models help scientists to estimate changes in yields and other parameters. The final yields capture the influence of GCMs, the interactions between soil, climate, and crop management (Islam et al. 2016). Bassu et al. (2014) reported that the multi-model approach is more efficient than one single model in determining mean yield while having very limited data for model calibration. In the current study, variations in the performance of five GCMs on maize yield along with three maize models (CERES-Maize, IXIM-Maize, and APSIM-Maize) were observed (Fig. 13). Spring season average maize yield is expected to decline 440 kg ha^−1^, 770 kg ha^−1^, 1538 kg ha^−1^, and 2047 kg ha^−1^, predicted by middle, hot wet, cool dry, and hot dry climate scenarios (Fig. 12). The decline in yield might be due to the phenomenon reported by Bassu et al. (2014) that high temperatures increased the development rate as a result of the duration of growth and developmental phases reduced. All three crop models (CERES-Maize, IXIM-Maize, and APSIM-Maize) predicted up to an 8–55% yield decline from baseline yield in spring yield during mid-century (Fig. 13). This decline in yield would be due to an increase in temperature. The CERES-Maize model showed 6–8% average increase in yield, while IXIM and APSIM-Maize presented an increase of yield from 8–19% during mid-century under cool wet climate scenarios (Fig. 13). These results are in line with the results of Dimes et al. (2003) and Chen et al. (2012) which reported that APSIM performed well under changing climate scenarios. Maize yield may slightly increase from the baseline under sub-humid climate whereas decreased under semi-arid conditions (Araya et al. 2015). Pandey et al. (2007) noticed that rising temperature had reduced (8 to 31%) the maize yield by using DSSAT. The Agricultural Model Inter-comparison and Improvement Project (AgMIP) maize model inter-comparison project studies found large variations among multiple maize model yield predictions (Bassu et al. 2014). Climate change impacts on maize yields for most important climate scenarios range between −20 and −45% for maize by 2100 (Müller and Elliott 2015). The above-mentioned decline in yield is due to increased temperature and less rainfall causing huge losses to the farming community.

Adaptation strategies are necessary to cope up with the hazardous effects of climate change in the future. Modifying the management practices such as sowing dates, varying fertilizer rates, irrigation applications (Figs. 14 and 15) and enhancing the genetic potential of cultivars could be beneficial for increasing maize productivity in future climate change scenarios. Maize crop models (CERES-Maize, CSM-IXIM-Maize, APSIM-Maize) predicted higher net return in terms of yield (kg ha^−1^) when maize is planted at sowing date (30 January) for all five GCMs during mid-century RCP 8.5. Fifteen (15) days earlier sowing of maize has a bigger yield potential than the current sowing time (mid-February). It could be a better planting time during mid-century than other planting dates (Fig. 14). While at this planting date (30 January) in combination with 20% more fertilizer and 90% efficiency of irrigation practice can give 20–30% higher yield than the current sowing time (Fig. 15). The crop production enhanced when the rate of nitrogenous fertilizer increased from the recommended as presented in previous studies (Wajid et al. 2010). Similarly, Deb et al. (2014) reported that by modifying the sowing dates, then the current dates may increase yield from 5 to 22.5% in the future. It might be due to an extension in the growing and grain filling period when planted earlier than the current planting date as mentioned by Lv et al. (2019). Amin et al. (2018) recommend the moderate use of fertilizer doses to avoid the potential threats of a future changing climate. Further, there is a need to test adaptation technology in the field and controlled conditions by providing future climate projections.

## Conclusions

Changing climate adversely affects agricultural productivity and creates food insecurity. Crop growth models are modern and efficient tools that have been extensively used in mounting the climate change impacts and developing adaptation packages for sustainable crop production under changing climate. Based on the findings of this study, it is concluded that all three models (CERES-Maize, IXIM-Maize, and APSIM) performed reasonably well for spring plantation and could be used under arid to semi-arid climatic conditions. Simulating behavior and performance of CERES-Maize and APSIM-Maize for phenological events was good, whereas CSM-IXIM-Maize simulations for leaf area index, biomass, and yield were found well during both studied years. Keeping in view the climate change impacts, there would be a rise in temperature 1.57–3.29 °C and variations in rainfall during maize growing season in all five GCMs with RCP 8.5 scenarios for the mid-century (2040–2069). Maize models (CERES-Maize, IXIM-Maize, and APSIM-Maize) predicted up to an 8–55% yield decline from baseline yield for all GCMs in spring yield during mid-century. In the spring season, early plantations may be beneficial to get a higher yield. Hybrid FH-1898, being a late-maturing hybrid, will get the benefit for yield when sown earlier in spring. P-1543 and 30-Y-87 are short-duration and high-yielding hybrids for the current production system, respectively. Climate change is negatively affecting current and future maize production systems, but these negative effects on crop production can be minimized through following management and adaptation strategies like modifying the planting dates (30 January in spring than mid-February) and 20% increase in nitrogen fertilizer, together with 90% irrigation (fertigation) and development of heat and drought-tolerant hybrids.

## Data Availability

The datasets used and/or analyzed during the current study are available from the corresponding author on reasonable request.
